# Knowledge, Attitudes, and Practices of Breast Milk Feeding Among Mothers of Very Low Birth Weight Infants: A Study From Two Tertiary Centers

**DOI:** 10.7759/cureus.95900

**Published:** 2025-11-01

**Authors:** Aisha Fadhilah Abang Abdullah, Nadiah Azlisham, Mohd Fahmi Ellias, Melissa A Nunis, Farah Inaz Syed Abdullah, Nurul Husna Mohd Shukri, Zurina Zainudin

**Affiliations:** 1 Department of Paediatrics, Faculty of Medicine and Health Sciences, Universiti Putra Malaysia, Selangor, MYS; 2 Department of Paediatrics, Hospital Tunku Azizah, Kuala Lumpur, MYS; 3 Department of Nutrition, Faculty of Medicine and Health Sciences, Universiti Putra Malaysia, Selangor, MYS

**Keywords:** attitude, breastmilk feeding, knowledge, practice, very low birth weight

## Abstract

Background: Breastfeeding is advocated as optimal for very low birth weight (VLBW) infants. However, initiating breastfeeding presents significant challenges due to their immature suckling and fragile health status. This study evaluated maternal knowledge, attitude, and breast milk expression practices among mothers of VLBW infants.

Methods: A descriptive cross-sectional study was conducted in two Neonatal Intensive Care Units located in the Klang Valley, Malaysia, from March to December 2020. Mothers of VLBW infants were included once their infants achieved full enteral feeding. Data were collected using a self-reported questionnaire that incorporated sociodemography, breastfeeding knowledge, attitudes, and practices (KAP). Descriptive statistics were used to summarize participant characteristics, while Spearman’s correlation analysis examined the relationships among KAP variables. Statistical significance was set at *P* < 0.05.

Results: A total of 111 mothers participated, with the majority demonstrating good knowledge (mean score 9.20 ± 0.90) and neutral attitudes toward breastfeeding (mean score of 61.76 ± 4.81). Although more than half (60, 54.1%) initiated breast milk expression within 24 hours after delivery, only 30 (27%) produced colostrum within this timeframe, reflecting delayed lactogenesis associated with preterm delivery and maternal-infant separation. Despite a moderate positive correlation between maternal knowledge and attitudes toward breastfeeding (Spearman’s rho = 0.41, *P* < 0.001), only 31 out of 111 mothers (27.9%) provided sufficient expressed breast milk until their VLBW infants achieved full enteral feeding.

Conclusions: Despite VLBW mothers exhibiting adequate knowledge about breastfeeding and acceptable attitudes towards breastmilk feeding, actual practices are suboptimal. Identifying barriers, improving healthcare protocols, and strengthening the family support system are essential steps to enhance breastfeeding rates and improve health outcomes for VLBW infants.

## Introduction

Very low birth weight (VLBW) infants, defined as those weighing less than 1,500 g at birth, face significant challenges arising from physiological immaturity and contribute disproportionately to neonatal morbidity and mortality worldwide [1,2]. In Malaysia, data from the Malaysia National Neonatal Registry (MNNR) indicate that in 2020, across 46 participating hospitals with 319,867 live births, 10.7% were delivered preterm (<37 weeks); within the registry cohort (*n* = 20,033) that met predefined clinical inclusion criteria, 3,768 infants (18.8%) had birth weights ≤1,500 g, corresponding to approximately 1.2% of live births in participating hospitals captured as VLBW that year [2]. These infants are particularly vulnerable to complications such as respiratory insufficiency, patent ductus arteriosus, intraventricular hemorrhage, necrotizing enterocolitis, and sepsis, leading to adverse neurodevelopmental outcomes, prolonged hospitalization, greater healthcare costs, and psychological strain for families [3,4].

Over recent years, advances in perinatal and neonatal care have improved the survival and long-term outcomes of VLBW infants [4,5]. Within this context, optimal nutrition during the early postnatal period has emerged as a critical determinant of growth and neurodevelopment. Adequate nutrient intake and growth velocities approximating intrauterine rates have been associated with better neurodevelopmental scores, higher intelligence quotient, and lower rates of cerebral palsy [6,7]. Breastmilk is universally acknowledged as the best source of nutrition for all infants and is particularly vital for VLBW infants. Beyond providing essential macronutrients, breastmilk offers immunological protection, bioactive factors, and easily digestible fats that meet the unique needs of premature infants [8]. The World Health Organization (WHO) strongly recommends exclusive feeding with mother’s own milk for all low‑birth‑weight infants, including VLBW infants, for the first six months of life [9].

Despite these benefits, direct breastfeeding in VLBW infants is often limited due to immature sucking reflexes, gastrointestinal immaturity, and the need for intensive medical support [10]. In the NICU setting, mother-infant separation means that feeding in this group relies on expressed milk delivered via orogastric tube. Practice-related factors such as early initiation of expression, high frequency of expression during the first few days, and higher milk volumes as associated with greater breastmilk provisions in VLBW infants; conversely, being extremely premature and having intrauterine growth restrictions are the barriers [11]. Despite written information, most mothers still report gaps in guidance and support for the expression, storage, and transportation of breastmilk during the period of separation from their babies [12]. Unfortunately, many still rely on non-clinical information sources and require lactation support by the time the infants are ready to be started on breastfeeding [13].

Previous studies have highlighted that limited awareness, cultural perceptions, maternal anxiety, and lack of support for healthcare providers can hinder breastfeeding success in this vulnerable group [11,14]. Hence, the successful initiation and continuation of breastmilk feeding among VLBW infants depend heavily on maternal knowledge, attitudes, and practices (KAP) related to breastmilk expressing. Adequate knowledge enables apt initiation and sustained breastmilk expression; positive attitudes foster motivation and confidence, while appropriate practices ensure consistent provision of expressed breastmilk despite challenges such as prolonged hospitalization or maternal stress. Understanding the KAP of mothers with VLBW infants is essential to enhance and promote exclusive breastmilk feeding, optimize infant health outcomes, and support maternal well-being. However, there is limited evidence on maternal KAP related to breast milk feeding among mothers of VLBW infants in Malaysia. Therefore, this study was conducted to assess the KAP of breast milk feeding among mothers of VLBW infants in two tertiary centers in the Klang Valley, Malaysia.

## Materials and methods

This descriptive cross-sectional study was conducted at two tertiary-level Neonatal Intensive Care Units (NICUs) within the Klang Valley region between May and December 2020. The original study had dual objectives: (a) to identify factors associated with successful breastmilk feeding among VLBW infants, and (b) to assess KAP regarding breastmilk feeding in the same population. Both centers serve as major referral hospitals for high-risk neonatal cases and high patient volume, standardized care protocols, and the capacity to provide comprehensive data aligned with the study objectives. A consecutive, non-probability sampling method was applied and recruit all mothers of VLBW infants admitted to the participating NICUs during the study period. Mothers were approached once their infants had achieved full enteral feeding, defined as a minimum intake of 120 mL/kg/day. Inclusion criteria were mothers of VLBW infants admitted to NICU who could understand and read English or the local (Malay) language, and consented. Those who were illiterate, had underlying chronic medical illnesses (e.g., heart failure, chronic renal failure, malignancy, mental illnesses), or whose infants had dysmorphic features and/or lethal congenital anomaly, fatal congenital conditions, or were severely ill and succumbed before two weeks of age were excluded. These exclusion criteria were applied to ensure the accuracy of responses and minimize potential confounding factors. Successful establishment of breastmilk feeding was defined as infants exclusively receiving expressed breast milk from their mothers from the initiation of feeding until achieving full enteral feeding. 

Sample size calculation

The required sample size was determined using the single population proportion formula described by Lwanga and Lemeshow [15]. Assuming a proportion of good breastfeeding practice of 50%, a 95% confidence level (*Z* = 1.96), and a margin of error of 10%, the minimum calculated sample size was 96 participants. To account for a potential 20% non-response rate, the final target sample size was increased to 122 mothers. Only a total of 111 eligible mothers were available during the study period; all completed the study with no dropouts.

Questionnaire and scoring

A comprehensive literature review identified appropriate items for the study instrument. A final 38-item self-administered breastmilk feeding KAP questionnaire was developed as the study instrument. The questionnaire had two main components: (1) sociodemographic and clinical characteristics of the mother and infants, and (2) three sections on KAP (Appendices A-C). Section A was a 6-item scale designed to assess breast milk expression practices, including the time (in days) of first initiation of breast milk expression, the time (in days) when the mother first produces colostrum, the frequency of breast milk expression per day and the duration of breast milk expression per session as well as sentiments that would impede milk expressions (tiredness and painful milk expression). Each questions have two options and is coded as *1* or *0,* whereby a higher score reflects better practice, and scores range from 0 to 6. Section B was assessed using a questionnaire adapted from a KAP study conducted in India by Vijayalakshmi et al. [16], which consisted of a 15-item maternal breastmilk feeding knowledge questionnaire; each question had two options: *True* or *False*, with a total score ranging from 0 to 15. Higher scores indicated better breastmilk feeding knowledge. In Section C, the maternal attitude towards breastfeeding was assessed using a 17-item scale Iowa Infant Feeding Attitudes Scale (IIFAS) [17]. This validated instrument measured maternal attitude towards infant feeding methods and predicted breastfeeding intention and exclusivity. The IIFAS employs a 5-point Likert scale ranging from *strongly disagree* to *strongly agree*, with nine negatively worded items (reverse-coded). The score ranges between 17 and 85, with higher scores indicating more positive attitudes toward breastfeeding. The questionnaires were translated into Malay by a bilingual language expert, ensuring linguistic and cultural appropriateness for the local population. Permission to use the original knowledge questionnaire and the IIFAS for the attitude domain was obtained from the respective developers. 

Validity and reliability

The translated questionnaire underwent content validation by a panel of experts comprising two neonatologists, three pediatricians, and five senior nurses. Content validation yielded acceptable item-level content validity index (I-CVI) values of 0.8 to 1, with scale-level content validity index for universal agreement (S-CVI/UA) of 1, 0.9, and 0.88 for Sections A, B, and C, respectively. The average content validity index (S-CVI/Ave) was 1.00 for Section A and 0.98 for Sections B and C. Section A was self-developed by the research team based on relevant literature and expert consultation. Section B consisted of items adapted from a previously published source [16]. Despite attempts to contact the original authors, additional reliability information could not be obtained. Section C was IIFAS, which demonstrated Cronbach’s alpha values of 0.68-0.86 in the original validation [17] and 0.62 in the previously validated Malay version** **[18]. Following expert review and necessary modification, the questionnaire set was pilot-tested with 30 randomly selected mothers of premature newborns to ensure clarity and comprehensibility. In the current study, the overall breastfeeding KAP questionnaire demonstrated acceptable internal consistency reliability (Cronbach's α = 0.703).

Data collection

Participants were briefed on the study’s objectives and provided with an information sheet before obtaining their written consent. Confidentiality measures were thoroughly explained and assured to participants. The estimated time for completion of the questionnaire was 5 to 10 minutes. Researchers were available in the vicinity should any question about questions arise. Completed questionnaires were then collected by the researcher for data input and analysis.

Statistical analysis

Descriptive data were presented as frequency and percentage, and continuous variables were reported as means and standard deviation, and/or median and interquartile range (IQR). The relationship between dependent and independent variables was tested by Pearson’s chi-square test. Responses of the negatively worded items were reversed before analysis. Finally, Spearman’s correlation analysis was performed to examine the relationships among KAP variables. Interpretation of hypothesis test based on correlation strength, correlation direction, and *P*-value: The strength of correlation (*r*) was reported as either: 0.00-0.1 = negligible correlation; 0.10-0.39 = weak correlation; 0.40-0.69 = moderate correlation; 0.70-0.89 = strong correlation; 0.90-1.00 = very strong correlation. *P*-value < 0.05 implies that there is a significant correlation between any two variables tested. The data were analyzed using statistical software IBM Statistical Package for the Social Sciences (SPSS) version 30.0 (IBM Corp., Armonk, NY).

## Results

A total of 111 mothers agreed to participate in this study, and the majority of the respondents (87/111, 78.4%) were 25 years old and above (Table 1). Malays contributed to the largest percentage of total respondents, 66/111 (59.5%), almost all (107/111, 96.4%) were married, half of them (60/111, 54.1%) were primigravida, and 98 of 111 (88.3%) respondents had a singleton pregnancy. A quarter of the respondents (27/111, 24.3%) had underlying chronic medical illness, and pregnancy-related complications occurred in about 55/111 (49.5%) respondents. The majority of the respondents (86/111, 77.5%) and their spouses (90/111, 81.1%) received secondary education, and more than half of them (78/111, 70.3%) were from the middle-income group with monthly household incomes ranging between RM3000 and RM6000. 

**Table 1:** Sociodemographic and clinical characteristics of study population (N = 111).

	Frequency (*n*)	Percentage (%)
Age (years)		
<25	24	21.6
≥25	87	78.4
Ethnicity		
Malay	66	59.5
Chinese	17	15.3
Indian	16	14.4
Others	12	10.8
Marital status		
Married	107	96.4
Single	4	3.6
Parity		
Primiparity	60	54.1
Multiparity	51	45.9
Type of pregnancy		
Singleton	98	88.3
Twin	12	10.8
Triplet	1	0.9
Pregnancy-related complications		
Yes	55	49.5
No	56	50.5
Underlying medical problems		
Yes	27	24.3
No	84	75.7
Mode of delivery		
SVD	50	45
LSCS	61	55
Education level		
Primary	2	1.8
Secondary	86	77.5
Tertiary	23	20.7
Husband`s education level		
Primary	2	1.8
Secondary	90	81.1
Tertiary	19	17.1
Household income		
	25	22.5
RM3000-RM6000	78	70.3
>RM6000	8	7.2

In this study, 31 (27.9%) out of 111 VLBW mothers provided sufficient expressed breast milk until their infants reached full enteral feeding. Maternal knowledge of breastfeeding was generally high across most domains examined (Table 2). All mothers (111/111, 100%) had correctly identified that colostrum is the first breast milk, recognized the importance of adequate nutrition, the need for comfort positioning during breastfeeding, and the role of mother-infant bonding. Near-universal knowledge was also reported for the importance of colostrum in immunity, burping after feed practice, and maintaining eye contact. However, the were knowledge gaps about maternal illness during breastfeeding, whereby nearly half (52/111, 46.8%) believed that breastfeeding should be discontinued when the mother has diarrhea. In general, the median knowledge score on breastfeeding for our respondents was 14.0 (IQR 13.0-15.0). The majority (107/111, 96%) of the mothers in our study had good knowledge of breastfeeding (score 8-10). For the attitude section, the majority of mothers chose be neutral attitude in more than half the items tested (Table 3). However, most of them agreed that breast milk is easily digested (108/111, 97.3%), ideal for the babies (110/111, 99.1%), and more convenient than formula feeding (94/111,84.7%). Conversely, a large number of mothers agreed on the statements that breast milk is lacking in iron (104/111, 93.6%) and that formula milk is as healthy as breast milk (93/111, 83.8%). The average attitude score was 61.76 ± 4.81. 

**Table 2:** Maternal knowledge toward breastfeeding (N = 111). *Negatively worded item.

Statement		Respondent’s answer	
		True *n* (%)	False *n* (%)
1	Colostrum is the first breast milk.	111 (100)	0 (0)
2	Colostrum is important for the baby to maintain immunity.	109 (98.2)	2 (1.8)
3	Burping should be done after each feed.	110 (99.9)	1 (0.1)
4	Breastfeeding should be continued up to two years.	108 (97.3)	3 (2.7)
5	Exclusive breast milk can be given during the first six months.	103 (92.8)	8 (7.2)
6	Lactating mothers should take healthy food to improve the secretion of milk.	111 (100)	0 (0)
7	During breastfeeding, the mother should sit comfortably.	111 (100)	0 (0)
8	During breastfeeding, the mother should maintain eye-to-eye contact and talk with the baby.	109 (98.2)	2 (1.8)
9	Wash each breast with warm water before breastfeeding.*	85 (76.6)	26 (23.4)
10	Awakening the baby while breastfeeding.	105 (94.6)	6 (5.4)
11	Breastfeeding helps in mother-child bonding.	111 (100)	0 (0)
12	Breastfeeding can prevent diseases affecting the breast.	108 (97.3)	3 (2.7)
13	Breastfeeding affects the beauty of breastfeeding mothers.*	103 (92.9)	8 (7.2%)
14	A mother should not breastfeed the child when she has diarrhea.*	59 (53.2)	52 (46.8)
15	Stop breastfeeding when you start weaning.*	98 (88.3)	13 (11.7)
	Knowledge score (median (IQR))	14.0 (13.0-15.0)	

**Table 3:** Maternal attitude toward breastfeeding (N = 111). *Reverse-scored item.

Statement	Strongly agree, *n* (%)	Agree, *n* (%)	Neutral, *n* (%)	Disagree, *n* (%)	Strongly disagree, *n* (%)
						The nutritional benefits of breast milk last only until the baby is weaned from breast milk.*	1 (0.9)	13 (11.7)	89 (80.2)	3 (2.7)	5 (4.5)
Formula feeding is more convenient than breastfeeding.*	23 (20.7)	54 (48.6)	27 (24.3)	3 (2.7)	4 (3.6)
Breastfeeding increases mother-infant bonding.	37 (33.3)	71(64.0)	1(0.9)	1(0.9)	1 (0.9)
Breast milk is lacking iron.*	51(45.9)	53 (47.7)	1(0.9)	2 (1.8)	4 (3.6)
Formula-fed babies are more likely to be overfed than are breastfed babies.	1(0.9)	8 (7.2)	77 (69.4)	22 (19.8)	3 (2.7)
Formula feeding is the better choice if a mother plans to work outside the home.*	11 (9.9)	16 (14.4)	72 (64.9)	6 (5.4)	6 (5.4)
Mothers who formula feed miss one of the great joys of motherhood.	7 (6.3)	1(0.9)	84 (75.7)	12 (10.8)	7 (6.3)
Women should not breastfeed in public places such as restaurants.*	4 (3.6)	15 (13.5)	81 (73.0)	1 (0.9)	10 (9.0)
Babies fed breast milk are healthier than babies who are fed formula.	29 (26.1)	65 (58.6)	6 (5.4)	9 (8.1)	2 (1.8)
Breastfed babies are more likely to be overfed than formula-fed babies.*	9 (8.1)	36 (32.4)	57 (51.4)	3 (2.7)	6 (5.4)
Fathers feel left out if a mother breastfeeds.*	9 (8.1)	10 (9.0)	91 (82.0)	0 (0)	1(0.9)
Breastmilk is the ideal food for babies.	40 (36.0)	70 (63.1)	0 (0%)	0 (0%)	1 (0.9%)
Breastmilk is more easily digested than formula.	40 (36.0)	68 (61.3)	1 (0.9)	1(0.9)	1 (0.9)
Formula milk is as healthy for an infant as breast milk.*	34 (30.6)	59 (53.2)	6 (5.4)	10 (9.0)	2 (1.8)
Breastfeeding is more convenient than formula feeding.	28 (25.2)	66 (59.5)	7 (6.3)	6 (5.4)	4 (3.6)
Breastmilk is less expensive than formula.	37 (33.3)	70 (63.1)	0 (0)	1(0.9)	3 (2.7)
A mother who drinks alcohol once a week should not breastfeed her baby.*	5 (4.5)	5 (4.5)	88 (79.3)	8 (7.2)	5 (4.5)
Attitude score (mean ± SD)	61.76 ± 4.81				

The majority of the mothers (60/111, 54.1%) successfully initiated breast milk expression within less than 24 hours after delivery; however, only 30 of them (27%) produced colostrum within the same timeframe. Furthermore, the majority of mothers (69/111, 62.2%) expressed breast milk for less than 20 minutes per session and did so at least 6 times a day. Consequently, the maternal breast milk expression practice, on the whole, was poor, as indicated by a mean score of 3.03 ± 1.84 (range 0 to 6) (Table 4). As the data were collected by day 14 post-delivery and the babies' weight remained <1,500 g, none of the mothers had started kangaroo care or skin-to-skin contact. From this study, bivariate analysis of the binary outcome (successful establishment of breast milk feeding/ not successful) did not reveal any significant association with knowledge, attitudes toward breastfeeding, and breast milk expression practices. A Spearman’s correlation analysis was conducted to evaluate the relationship between maternal knowledge, attitude, and practice scores. The findings revealed a moderate positive correlation between maternal knowledge and attitude scores, which proved to be statistically significant (rs(109) = 0.445, *P* < 0.001). This outcome suggests that an increase in maternal knowledge is associated with a more favorable attitude toward breast milk feeding. However, the correlation between maternal knowledge and practice, as well as attitude score and practice, did not demonstrate significance, with rs(109) = -0.134, *P* = 0.160 and rs(109) = -0.018, *P* = 0.855, respectively (Table 5).

**Table 4:** Maternal breast milk expression practices (N = 111).

Item	Frequency, *n* (%)
(1) Initiation of breast milk expression post-delivery	
<24 hours	60 (54.1)
≥24 hours	51 (45.9)
(2) First colostrum production post delivery	
<24 hours	30 (27.0)
≥24 hours	81(73.0)
(3) Duration of breast milk expression per session	
<20 minutes	69 (62.2)
≥20 minutes	42 (37.8)
(4) Frequency of breast milk expression a day	
<5 times	42 (37.8)
≥6 times	69 (62.2)
(5) Expressing breastmilk is tiring	
Yes	43 (38.7)
No	68 (61.3)
(6) Expressing breastmilk is painful	
Yes	44 (39.6)
No	67 (60.4)
(7) Practice score (mean ± SD)	3.03 ± 1.84

**Table 5:** Correlation between knowledge, attitude, and practices of breastmilk feeding among mothers of premature infants (N = 111). *P* < 0.05 was considered statistically significant. *Significant *P*-value.

Correlation between	Spearman’s ρ	*P*-value	Inference
Knowledge and attitude	0.455	<0.001*	Moderate positive correlation
Knowledge and practice	-0.134	0.16	No significant correlation
Attitude and practice	-0.018	0.855	No significant correlation

## Discussion

In our study, the vast majority of mothers (107, 96%) exhibited a commendable level of knowledge regarding breastfeeding, with a median score of 14.0 (IQR 13.0-15.0). While this reflects positively on maternal health education efforts in our healthcare setting, possessing good knowledge about exclusive breastfeeding alone does not necessarily translate into practice. Only 31 mothers (27.9%) in our cohort successfully provided adequate breast milk to their VLBW infants until they achieved full enteral feeding, a rate consistent with findings from other studies [19-21]. Therefore, a comprehensive intervention is imperative to enhance breastfeeding rates in VLBW infants. Holistic postnatal breastfeeding education coupled with emotional support has been shown not only to increase maternal knowledge but also to be associated with significantly higher rates of exclusive breastfeeding at discharge [21,22]. Additionally, it is essential to explore sociocultural influences and maternal perceptions regarding breastfeeding within the community [23,24].

Besides possessing a strong understanding of breastfeeding, most of the mothers had a moderate attitude towards breastfeeding; the mean attitude score was 61.76 ± 4.81. Previous studies have highlighted the importance of a positive attitude toward breastfeeding as an independent, robust predictor for exclusive breastfeeding [25,26]. Mothers with a favorable attitude toward breastfeeding are more likely to exclusively breastfeed compared to those with a negative attitude [26]. Premature births often hurt maternal emotional well-being and parental self-image. Feelings of guilt, anxiety, and stress, particularly stemming from early infant-mother separation when infants are admitted to the NICU, can influence maternal attitudes and decisions regarding breastfeeding. Mothers may perceive themselves as incapable of providing sufficient breast milk to support the optimal growth of their VLBW infants [27]. Therefore, to foster a more positive outlook on exclusive breast milk feeding, it is imperative to develop and implement strategies that address the psychological needs of mothers of VLBW infants. Healthcare professionals should focus on refining antenatal and postnatal breastfeeding counseling sessions and providing mothers with robust emotional support. A study in the USA has reported that mothers who received encouragement from the hospital staff exhibited more positive attitudes toward pumping and expressed greater intention to breastfeed their VLBW infants, which significantly correlated with a higher rate of maintaining breast milk supply up to 48 weeks’ corrected age [28].

Our study found no association between maternal breast milk expression practices and successful breast milk feeding among VLBW infants. This contrasts with findings from other studies indicating that early initiation of breast milk expression is associated with increased milk production and earlier onset of lactogenesis stage II [29]. Furman et al. [30] observed that early initiation of breast milk expression within the first six hours post-delivery, coupled with frequent milk expression (at least five times per day), was associated with a higher likelihood of mothers maintaining lactation beyond 40 weeks’ corrected age. These positive correlations persisted even after adjusting for maternal age, race, marital status, and education beyond high school. The authors suggested increased maternal support, particularly targeted at behavioral factors such as early and frequent milk expression and kangaroo care, may enhance successful lactation rates among mothers of VLBW infants who opt to breastfeed. Delayed initiation of breast milk expression also demonstrated a dose-response effect, with later initiation associated with higher odds for inadequate breastfeeding duration [31]. Despite consistent evidence indicating that early initiation and regular expression of breast milk have a positive impact on successful breastfeeding among premature infants, our study did not yield similar results. This discrepancy may be attributed to the small sample size, highlighting the need for a larger sample to explore this factor, especially considering that our data indicated a trend toward significant factors affecting the successful establishment of breast milk feeding.

Although our study identified a significant correlation between knowledge and attitude, this did not translate into good breast milk feeding practices. Known barriers and challenges to establishing breast milk feedings include coexisting medical conditions in premature infants, maternal intention, and perceived support. Despite having robust breastfeeding policies, the implementation of such practices often faces challenges, such as delays in initial postpartum contact with the unit’s breastfeeding team, consequently delaying the initiation of breast milk expression. Some mothers may take more than a day to express breast milk, particularly in cases of post-cesarean section or when mothers require ICU/HDU care for underlying medical illness or pregnancy-related complications. Some mothers find breast milk expression painful and exhausting, making adherence to the routine challenging. Others may encounter difficulties with storing breast milk or transporting it to the NICU. Additionally, parity and lack of experience in expressing breast milk play pivotal roles in the successful establishment of breast milk feeding. More than half of the mothers (54.1%) in our study were primigravida and lacked experience in breastfeeding, let alone expressing breast milk for their VLBW infants. Other factors that may contribute to our findings, albeit not assessed in this study, include mothers’ initial intention to breastfeed, attendance at antenatal classes before delivery, volume per expression during the first two weeks of life, maternal smoking, and maternal psychological behavior. In our local NICU setting, the majority of VLBW infants are initiated on enteral feeding within 24 hours of life. If mothers fail to initiate early expression of breast milk and experience delays in producing sufficient colostrum, their infants are more likely to receive formula milk.

This study comprehensively examines breastfeeding KAP among mothers of VLBW infants. However, the study is constrained by its small sample size, which may limit the generalizability of findings and the ability to detect significant associations. Additionally, certain influential factors, such as maternal intention to breastfeed and attendance at antenatal classes, were not assessed, potentially leaving gaps in understanding. Furthermore, it relies on self-reported data from mothers, introducing the possibility of bias, particularly regarding breastfeeding practices. Despite these limitations, the study integrates previous research findings and advocates for a holistic intervention approach, emphasizing the importance of refining breastfeeding counseling and providing emotional support to mothers of VLBW infants.

## Conclusions

Despite the implementation of baby-friendly hospital initiatives, the prevalence of breast milk feeding establishment among VLBW infants remains alarmingly low, with less than one-third of mothers achieving successful establishment of breast milk feeding. Although mothers generally exhibit favorable knowledge and attitudes towards breastfeeding, the practice falls short of optimal. The Baby-friendly Hospital Initiative policy may raise awareness without corresponding improvement in practice, highlighting the need for more efforts to identify barriers faced by both mothers and health care professionals in the NICU setting. Evaluating quality improvement projects, clinical guidelines, hospital protocols, and other initiatives by healthcare providers, policymakers, and public health organizations presents a vital opportunity to improve breast milk feeding rates among VLBW infants and enhance their health outcomes. 

## Appendices

Appendix A 

**Table 6:** Practice domain of the KAP questionnaire Items created by the authors.

Statement	Answer
1. How soon after delivery did you try to express your breast milk?	In less than 24 hours
	more than 24 hours
2. How long did it take for your breast milk to come in for the first time?	In less than 24 hours
	more than 24 hours
3. Generally, how long did it take for one session of breast milk expression.	In less than 20 minutes
	more than 20 minutes
4. How frequent do you do breast milk expression within 24-hour period?	less than or equal 5 times
	more than 6 times
5. Do you think that expressing or pumping breast milk was too tiring?	Yes
	No
6. Did you experience any pain during breast milk expression?	Yes
	No

Appendix B 

**Table 7:** Knowledge domain of KAP questionnaire Reproduced with permission from Vijayalakshmi et al. (2015) [16].

Statement	Answer	
1. Colostrum is first breast milk	True	False
2. Colostrum is important for the baby to maintain immunity	True	False
3. Burping should be done after each feed	True	False
4. Breast feeding should be continued up to 2 years	True	False
5. Exclusive breast milk can be given during first 6 months	True	False
6. Lactating mother should take healthy food to improve secretion of milk	True	False
7. During breastfeeding the mother should sit comfortably	True	False
8. During breastfeeding the mother should maintain eye to eye contact and talk with the baby	True	False
9. Wash each breast with warm water before breast feeding*	True	False
10. Awakening the baby while breastfeeding	True	False
11. Breast feeding helps in mother and child bonding	True	False
12. Breast feeding can prevent diseases affecting breast	True	False
13. Breastfeed affect the beauty of feeding mothers*	True	False
14. Mother should not breastfeed the child when she has diarrhea*	True	False
15. Stop breastfeeding when you start weaning*	True	False
*negatively worded item		

Appendix C 

**Table 8:** Attitude domain of the KAP questionnaire from the Iowa Infant Feeding Attitude Scale (IIFAS) Reproduced with permission from Mora et al. (1999) [17].

Items / Score	STRONGLY DISAGREE	DISAGREE	NEUTRAL	AGREE	STRONGLY AGREE
1. The nutritional benefits of breast milk last only until the baby is weaned from breastmilk*					
2. Formula feeding is more convenient than breastfeeding*					
3. Breastfeeding increases mother-infant bonding					
4. Breastmilk is lacking iron*					
5. Formula-fed babies are more likely to be overfed than are breastfed babies					
6. Formula feeding is the better choice if a mother plan to work outside the home*					
7. Mothers who formula feed miss one of the great joys of motherhood					
8. Women should not breastfeed in public places such as restaurants*					
9. Babies fed breast milk are healthier than babies who are fed formula					
10. Breastfed babies are more likely to be overfed than formula fed babies*					
11. Fathers feel left out if a mother breastfeeds*					
12. Breastmilk is the ideal food for babies					
13. Breastmilk is more easily digested than formula					
14. Formula milk is as healthy for an infant as breast milk*					
15. Breastfeeding is more convenient than formula feeding					
16. Breastmilk is less expensive than formula					
17. A mother who drinks alcohol once a week should not breastfeed her baby					
* Reverse scored item					
